# Novel tRNA Synthetase Inhibitors Increase Healthspan, Lifespan, and Autophagic Flux in *C. elegans*

**DOI:** 10.3390/biom16010073

**Published:** 2026-01-01

**Authors:** Olivia C. Heath, Madison P. Otero, Alexander T. Achusim, Dorothy S. Karr, Antonio W. Leger, Mark A. McCormick

**Affiliations:** 1 Department of Biochemistry and Molecular Biology, School of Medicine, University of New Mexico Health Sciences Center, Albuquerque, NM 87131, USA; 2 Autophagy, Inflammation, and Metabolism Center of Biomedical Research Excellence, Albuquerque, NM 87131, USA

**Keywords:** Gcn4, ATF-4, ATF4, tRNA synthetase, autophagy, healthspan

## Abstract

We previously demonstrated that the tRNA synthetase inhibitors mupirocin and borrelidin extend lifespan in *C. elegans* and *S. cerevisiae* and that tRNA synthetase inhibition enhances autophagy in mammalian cells. In this study, we identify four additional tRNA synthetase inhibitors, REP8839, REP3123, LysRS-In-2, and halofuginone, that extend both healthspan and lifespan in *C. elegans*. These compounds also trigger a significant upregulation of autophagy, specifically at their lifespan-extending doses. These phenotypes partially depend on the conserved transcription factor ATF-4. Our findings further establish tRNA synthetase inhibition as a conserved mechanism for promoting increased lifespan and now healthspan, with potential implications for therapeutic interventions targeting age-related decline in humans.

## 1. Introduction

Aging has a profound impact on individuals, societies, and economies worldwide. As the proportion of older adults grows rapidly, so does the economic burden that comes with inevitable age-related diseases. The need for interventions designed to delay the onset of these age-related diseases has never been greater.

*C. elegans* has long been used as a model for aging research [1,2,3,4]. They have a relatively short lifespan of two to three weeks, making them ideal for studying the many factors that influence aging. Additionally, among the over 18,000 *C. elegans* protein sequences, at least 83% have human homologs [5]. This makes *C. elegans* a fast and efficient model organism to study aging and age-related diseases.

Macroautophagy, henceforth referred to as autophagy, is a cellular process that, in part, can act to break down damaged, dysfunctional, or otherwise unwanted components. Autophagy is crucial for maintaining proteostasis and is a necessary system for cellular survival under stressful conditions. Autophagic efficiency declines during aging, leading to the buildup of damaged proteins and organelles, as well as other nonviable cellular debris.

The amino acid response (AAR) pathway is a highly conserved mechanism that ultimately leads to the increased translation of Gcn4 (in yeast), ATF-4 (in worms), and ATF4 (in mammals). We have previously shown that activation of this pathway through the chemical inhibition of tRNA synthetases (tRS) can extend lifespan in both worms and yeast [6]. tRNA synthetases play a critical role in attaching amino acids to their corresponding tRNA, a process necessary for translation. When tRS enzymes are inhibited, uncharged tRNA accumulates. This response is sensed by the uncharged tRNA sensor, general control non-depressible kinase 2 (Gcn2 in yeast, GCN-2 in worms, and GCN2 in mammals).

The accumulation of uncharged tRNA prompts GCN2 to phosphorylate eukaryotic initiation factor 2 (eIF2) [7], which inhibits translation re-initiation until an eIF2 phosphatase restores eIF2’s function [8]. This delay in re-initiation leads to a global reduction in protein synthesis. Still, it paradoxically enhances the translation of certain genes, including the transcription factor ATF4 [9] (or its orthologs like Gcn4 and ATF-4), which regulates hundreds of target genes. ATF4 and its orthologs are controlled at the translational level by upstream open reading frames [10], a mode of regulation that is evolutionarily conserved. This suggests that ATF4 and its counterparts function as a conserved stress response system for translational disruption. However, the full downstream effects of tRS inhibition on this system remain largely undefined. Previous research in our lab has demonstrated that tRS inhibitors activate the AAR and extend lifespan in worms [6] and yeast, a process that is entirely dependent on Gcn4/ATF-4. This finding suggests a strong link between this conserved transcription factor and the aging process.

## 2. Materials and Methods

### 2.1. C. elegans Strains

All worm strains were maintained at 20 °C on NGM plates (Fisher FB0875714, Thermo-Fisher, Waltham, MA, USA) containing *E. coli* strain OP-50 as a food source [11]. Wild-type N2 worms, *atf-4* deletion worms (RB790 *atf-4(ok576)*), and autophagy reporter worms (MAH215 sqIs11 [*lgg-1*p::mCherry::GFP::*lgg-1* + *rol-6*]), as well as *E. coli* strain OP-50, were obtained directly from the *Caenorhabditis* Genetics Center (https://cgc.umn.edu/), URL accessed on January 15 2025, Minneapolis, MN, USA which is funded by NIH Office of Research Infrastructure Programs (P40 OD010440).

### 2.2. C. elegans Lifespan Experiments

**Figure 1:**
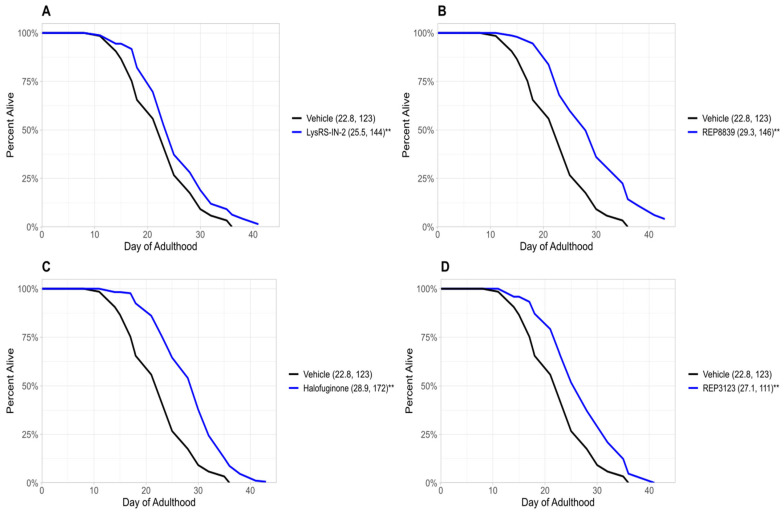
Novel tRNA synthetase inhibitors increase lifespan in wild-type *C. elegans*. (**A**) A total of 10 μM LysRS-IN-2 increases lifespan in wild-type *C. elegans* (blue) compared to the vehicle-treated group (black). (**B**) A total of 60 μM REP8839 increases lifespan in wild-type *C. elegans* (blue) compared to the vehicle-treated group (black). (**C**) A total of 1 μM halofuginone increases lifespan in wild-type *C. elegans* (blue) compared to the vehicle-treated group (black). (**D**) A total of 40 μM REP3123 increases lifespan in wild-type *C. elegans* (blue) compared to the vehicle-treated group (black). Vehicle = DMSO. ** *p* < 0.001. Values in parentheses indicate mean lifespan (days) and number of animals analyzed. *p* values were calculated using the log-rank (Mantel–Cox) test.

**Figure 2:**
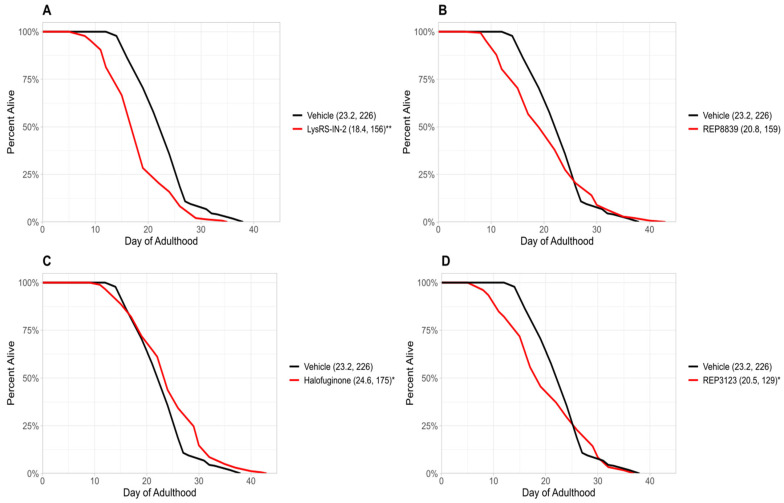
tRNA synthetase inhibitors do not increase lifespan in *atf-4(ok576) C. elegans*. (**A**) A total of 10 μM LysRS-IN-2 does not increase lifespan in *atf-4(ok576) C. elegans* (red) compared to the vehicle-treated group (black). (**B**) A total of 60 μM REP8839 does not increase lifespan in *atf-4(ok576) C. elegans* (red) compared to the vehicle-treated group (black). (**C**) A total of 1 μM halofuginone does not increase lifespan in *atf-4(ok576) C. elegans* (red) compared to the vehicle-treated group (black). (**D**) A total of 40 μM REP3123 does not increase lifespan in *atf-4(ok576) C. elegans* (red) compared to the vehicle-treated group (black). Vehicle = DMSO. * *p* < 0.01, ** *p* < 0.001. Values in parentheses indicate mean lifespan (days) and number of animals analyzed. *p* values were calculated using the log-rank (Mantel–Cox) test.

Lifespan analysis was conducted at 20 °C unless otherwise stated [12]. Specifically, all lifespans were carried out on 6 cm plates (T3306, Tritech Research, Los Angeles, CA, USA) containing standard NGM media [11] using on-plate UV-killed bacterial food (*E. coli* strain OP-50 unless otherwise specified) that was first plated at 50 μL per lifespan plate in the center of each plate, allowed to grow for 72 h at 25 °C, and then treated to 3 × 9999 μm Joules ×100 using a UV Stratalinker 2400 (Stratagene, San Diego, CA, USA) on uncovered plates. In all lifespans, 0.1 mM FUDR (5-fluorodeoxyuridine) (518265, Bio-World, Dublin, OH, USA) was added to plates at late L4 stage in order to suppress development of progeny. All drug treatments were added to plates after UV treatment and before addition of eggs and allowed to equilibrate overnight to ensure even diffusion through the agar. Reported concentrations represent the final agar concentrations. Each day, all worms were scored for movement using a 6.7×–45× Trinocular Zoom Stereo Microscope with attached incandescent reflected transillumination base (ZM-2T-EB, AmScope, Irvine, CA, USA), and any dead worms were removed using a pick made from a 5.75 inch plain soda-lime glass Pasteur pipette handle (Thermo-Fisher, Waltham, MA, USA) and a platinum wire tip (Tritech Research, Los Angeles, CA, USA). Bagged, missing (crawled off), or exploded worms were noted and censored daily. Mean lifespan (in days) and the number of animals analyzed (n) are reported in parentheses in the figure legends. As a reference for baseline viability, *atf-4(ok576)* showed a lifespan comparable to wild type under vehicle conditions in our assays (see Figure 1 and Figure 2 legends for mean and n).

### 2.3. tRNA Synthetase Inhibitor Drug Concentrations

All tRNA synthetase inhibitor concentrations were selected based on pilot lifespan experiments and literature precedent, where available. For each compound, several concentrations were screened to identify the lowest dose that reproducibly extended lifespan without visible toxicity (e.g., developmental delay, death, or growth defects). The concentrations reported in this study represent those optimal, non-toxic doses. Compounds showing no further benefit or exhibiting toxicity at higher concentrations were not pursued further.

### 2.4. Autophagy Assays

MAH215 *C. elegans* were maintained at 20 °C on NGM plates containing *E. coli* strain OP-50 as a food source [11]. tRS-treated nematodes were grown on plates with their respective drug treatments. For imaging, *C. elegans* were mounted on 1% agarose pads and paralyzed with 10 mM levamisole in M9 prior to fluorescence imaging, following established protocols [13] (Fisher, Waltham MA, USA, AC187870100). Imaging was performed on an Olympus iX83 Yokogawa spinning disc confocal microscope (Olympus, Center Valley, PA, USA) in the UNM Comprehensive Cancer Center Fluorescence Microscopy and Cell Imaging Shared Resource, which is supported by UNM Comprehensive Cancer Center Support Grant NCI P30CA118100. Wild-type and *atf-4(ok576)* animals were assayed concurrently under identical conditions. Vehicle-only controls for *atf-4(ok576)* are shown in Supplementary Figure S1. Autophagy flux measurements were conducted using animals derived from a single synchronized cohort per experiment. Following synchronization by egg lay, individual animals were imaged and quantified on the same day for each strain and condition, with 13–16 animals analyzed per strain × condition × day. These measurements reflect multiple individual animals within one biological experiment.

### 2.5. Healthspan/Thrashing Assays

*C. elegans* were grown on NGM plates containing their respective drug treatment. For the thrashing assays, nematodes were placed on a glass slide (AmScope, Irvine, CA, USA) containing liquid M9 and were allowed to acclimate to the liquid for 1 min. Subsequently, body bends were counted manually under a microscope for 1 min. Thrashing assays were performed on animals obtained from a single synchronized population per experiment. Worms were synchronized by egg lay and maintained under identical conditions, and individual animals were scored on the same day for each strain and treatment. Approximately 8 individual animals were measured per strain × treatment × day. These measurements represent multiple animals assessed within a single biological experiment.

### 2.6. Quantification and Statistical Analysis

Lifespan curves were plotted using Kaplan–Meier [14] estimates using R 4.5.0 (R Foundation, Vienna, Austria). Statistical significance for lifespan analyses was determined using the log-rank (Mantel–Cox) test implemented via survival::survdiff() from package survival 3.7-0 in R 4.5.0 (R Foundation, Vienna, Austria). Thrashing and autophagy assays were analyzed using Student’s *t*-test [15] with Bonferroni correction in R 4.5.0 (R Foundation, Vienna, Austria).

### 2.7. Materials

Halofuginone was purchased from Ambeed, Buffalo Grove, IL, USA [CAS No. 64924-67-0]. Borrelidin was purchased from BioViotica, San Diego, CA, USA (BVT-0098). LysRS-IN-2 was purchased from GLPBio, Montclair, CA, USA (GC65058). REP3123 and REP8839 were purchased from Axon Medchem, Reston, VA, USA (1704, 1705). Mupirocin was purchased from BOC Sciences, Shirley, NY, USA (B0084-056590). Borrelidin and mupirocin were included as positive controls for lifespan extension, based on prior studies. Because their effects on healthspan and autophagy had not been examined previously, they were also tested here to determine whether their lifespan-extending activity is accompanied by improvements in these related phenotypes.

## 3. Results

### 3.1. Novel tRNA Synthetase Inhibitors Extend Lifespan in C. elegans in an Atf-4-Dependent Manner

Previous studies from our group have demonstrated that treatment with tRNA synthetase inhibitors can extend lifespan in *C. elegans* and *S. cerevisiae* [6]. In the present study, we identify four novel tRNA synthetase inhibitors that also extend lifespan in wild-type *C. elegans* (Figure 1). These novel lifespan-extending inhibitors include two methionyl tRNA synthetase inhibitors, REP8839 (Figure 1B) and REP3123 (Figure 1D), as well as the lysyl tRNA synthetase inhibitor LysRS-In-2 (Figure 1A) and the prolyl tRNA synthetase inhibitor halofuginone (Figure 1C). Given that we have previously shown that these compounds greatly upregulate ATF4 translation in mammalian cells [16] and that previously identified tRNA synthetase inhibitors depend on *atf-4* for lifespan extension in *C. elegans* [6], we next used an *atf-4(ok576)* deletion strain to ask whether the observed lifespan extensions depend on *atf-4*. To assess this, we treated *atf-4(ok576) C. elegans* with the same tRNA synthetase inhibitors and found no significant increase in lifespan (Figure 2). Under our assay conditions, *atf-4(ok576)* exhibited a baseline lifespan comparable to the wild type, as indicated by the vehicle control curves (e.g., N2 mean ≈ 23.2 days vs. *atf-4* mean ≈ 22.8 days). This supports that loss of *atf-4* does not inherently reduce viability under baseline conditions. The results of Figure 2 indicate that the lifespan extension induced by these tRNA synthetase inhibitions is *atf-4*-dependent. For each of these tRNA synthetase inhibitors, the lifespan extension in wild-type animals was dose-dependent, and the largest effect size is shown in Figure 1. The doses used for each compound correspond to the lowest concentrations that consistently extended lifespan without visible toxicity, as determined by pilot dose-range assays.

These findings extend our prior work by broadening the claim that the entire class of drugs, tRNA synthetase inhibitors, elicits a pro-longevity effect. Notably, although the four compounds target distinct aminoacyl-tRNA synthetases, they all converge on a common downstream genetic requirement for *atf-4*, highlighting a conserved transcription factor as a key mediator of lifespan extension. This finding is promising for the possibility of the use of these drugs in higher organisms with the goal of promoting longevity in humans.

Together, these data identify a new set of candidate small molecules that extend lifespan in an *atf-4*-dependent manner and provide further mechanistic support for tRNA synthetase inhibition as a viable, safe, and evolutionarily conserved longevity intervention.

### 3.2. tRNA Synthetase Inhibitors Extend Healthspan, Specifically Thrashing Activity, in C. elegans at the Same Doses That Extend Lifespan

Having shown that these four novel compounds can extend lifespan in *C. elegans*, we next asked whether they could also influence healthspan. For comparison, we included two previously characterized tRNA synthetase inhibitors—borrelidin and mupirocin—that we have previously shown to extend lifespan in *C. elegans* and yeast [6]. These compounds had not yet been tested for effects on healthspan or autophagy and thus served both as positive controls for lifespan extension and as comparators to evaluate whether previously known tRNA synthetase inhibitors similarly enhance functional and cellular maintenance phenotypes. Healthspan refers to the healthy, active period of life, underscoring a growing interest in interventions that do not solely delay mortality without delaying morbidity but that increase the healthy, productive period of life as well [17,18,19]. One way that healthspan can be measured in *C. elegans* is by quantifying thrashing rates. When placed into liquid, these animals thrash back and forth at a steady rate, and this rate decreases consistently with age in wild-type animals [20]. To evaluate whether tRNA synthetase inhibition improves healthspan, we used a thrashing assay to measure the frequency of body bends that *C. elegans* voluntarily makes when suspended in liquid.

**Figure 3:**
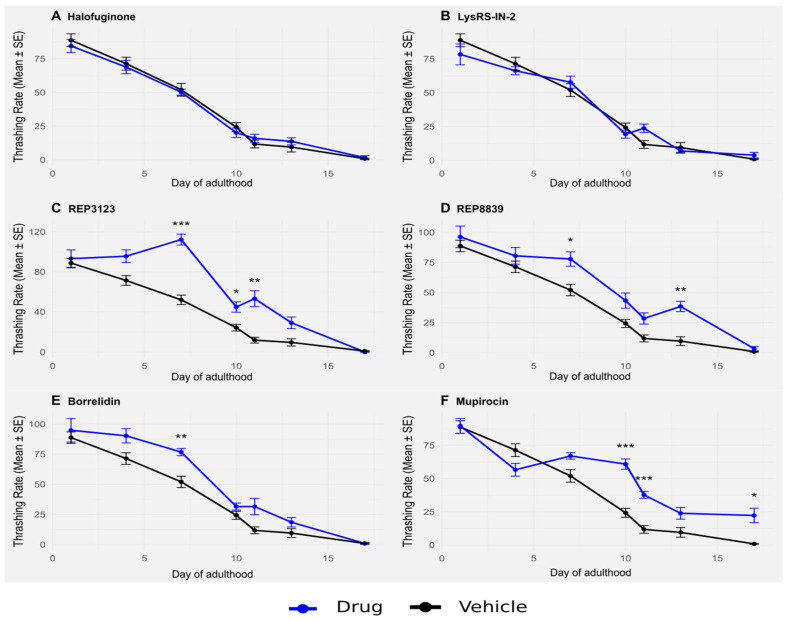
Thrashing in wild-type *C. elegans* is significantly improved upon treatment with some tRNA synthetase inhibitors. (**A**) A total of 1 μM halofuginone does not increase thrashing in wild-type *C. elegans* (blue) compared to the vehicle-treated group (black). (**B**) A total of 10 μM LysRS-IN-2 does not increase thrashing in wild-type *C. elegans* (blue) compared to the vehicle-treated group (black). (**C**) A total of 40 μM REP3123 significantly increases thrashing in wild-type *C. elegans* (blue) compared to the vehicle-treated group (black) on days 7, 10, and 11. (**D**) A total of 60 μM REP8839 significantly increases thrashing in wild-type *C. elegans* (blue) compared to the vehicle-treated group (black) on days 7 and 13. (**E**) A total of 75 μM borrelidin significantly increases thrashing in wild-type *C. elegans* (blue) compared to the vehicle-treated group (black) on day 7. (**F**) A total of 550 μM mupirocin significantly increases thrashing in wild-type *C. elegans* (blue) compared to the vehicle-treated group (black) on days 10, 11, and 17. * *p* < 0.05, ** *p* < 0.01, *** *p* < 0.001. *p* values calculated with Student’s *t*-test with Bonferroni correction (*n* = 7 comparisons per drug panel).

**Figure 4:**
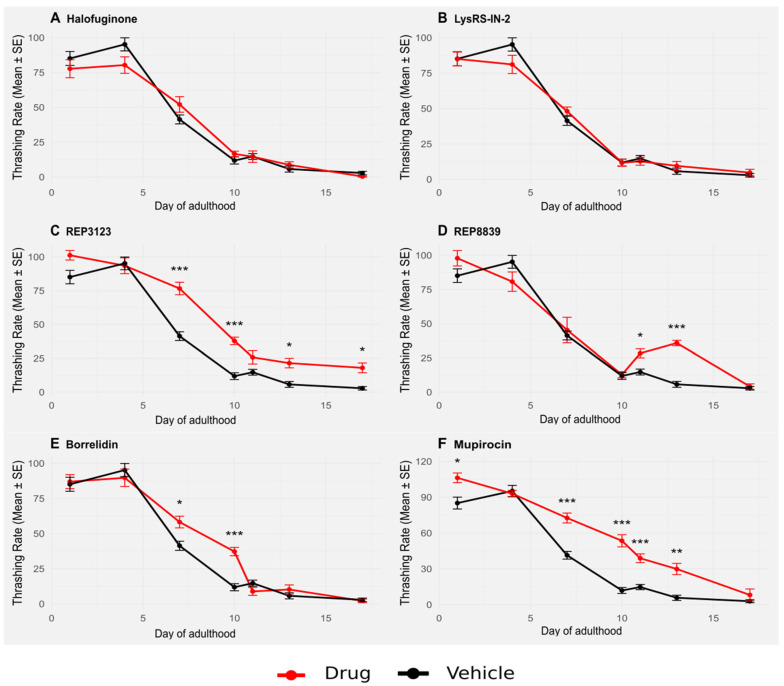
Thrashing in *atf-4(ok576) C. elegans* is significantly improved upon treatment with some tRNA synthetase inhibitors, although less so than the wild type. (**A**) A total of 1 μM halofuginone does not increase thrashing in *atf-4(ok576) C. elegans* (red) compared to the vehicle-treated group (black). (**B**) A total of 10 μM LysRS-IN-2 does not increase thrashing in *atf-4(ok576) C. elegans* (red) compared to the vehicle-treated group (black). (**C**) A total of 40 μM REP3123 significantly increases thrashing in *atf-4(ok576) C. elegans* (red) compared to the vehicle-treated group (black) on days 7, 10, 13, and 17. (**D**) A total of 60 μM REP8839 significantly increases thrashing in *atf-4(ok576) C. elegans* (red) compared to the vehicle-treated group (black) on days 11 and 13. (**E**) A total of 75 μM borrelidin significantly increases thrashing in *atf-4(ok576) C. elegans* (red) compared to the vehicle-treated group (black) on days 7 and 10. (**F**) A total of 550 μM mupirocin significantly increases thrashing in *atf-4(ok576) C. elegans* (red) compared to the vehicle-treated group (black) on days 1, 7, 10, 11, and 13. Vehicle = DMSO. * *p* < 0.05, ** *p* < 0.01, *** *p* < 0.001. *p* values calculated with Student’s *t*-test with Bonferroni correction (*n* = 7 comparisons per drug panel).

Wild-type *C. elegans* were treated with tRNA synthetase inhibitors at the same concentrations used to extend lifespan (Figure 1), and their thrashing rate was assessed throughout adulthood. Treatment with some of these compounds significantly enhanced thrashing, particularly later in life (Figure 3). Similar to lifespan extension, this improvement in healthspan was partially dependent on *atf-4* (Figure 4). In particular, animals treated with the compounds maintained higher thrashing rates at day 7 and beyond, a stage when untreated control animals typically show marked declines in movement and responsiveness. This preservation of neuromuscular function suggests that the benefits of tRNA synthetase inhibition are not limited to longevity alone but are accompanied by an overall delay in functional decline.

Importantly, these effects were consistent across multiple different tRNA synthetase inhibitors, indicating that healthspan extension may be a generalizable consequence of tRNA synthetase inhibition rather than an off-target effect of any individual compound. Moreover, the observation that *atf-4(ok576)* mutants showed less improved thrashing behavior in response to treatment reinforces the notion that this transcription factor plays a central role, although it is not solely responsible, in the protective response elicited by these compounds. Together, these results provide strong evidence that tRNA synthetase inhibitors not only extend lifespan but also maintain functional capacity during aging, an important goal of lifespan-extending therapies.

### 3.3. tRNA Synthetase Inhibitors Upregulate Autophagy at the Same Doses That Extend Lifespan

The effect of tRNA synthetases on lifespan that we have observed is dependent on *atf-4* (Figure 2). ATF-4 is itself a transcription factor that is translationally upregulated during the accumulation of uncharged tRNA [8,10,21]. Previous work has shown that upregulation of autophagy has been linked to increased lifespan [22], and we have previously shown that tRNA synthetase inhibitor treatment causes ATF-4-dependent changes in transcripts relating to autophagy in both mouse embryonic fibroblasts [16] and *S. cerevisiae* [23]. We have also shown that these tRNA synthetase inhibitors cause partially ATF-4-dependent increases in autophagic activity in mouse cells in vitro [16]. As a result, we wanted to ask whether autophagy was changed in *C. elegans* by lifespan-extending doses of tRNA synthetase inhibitors.

**Figure 5:**
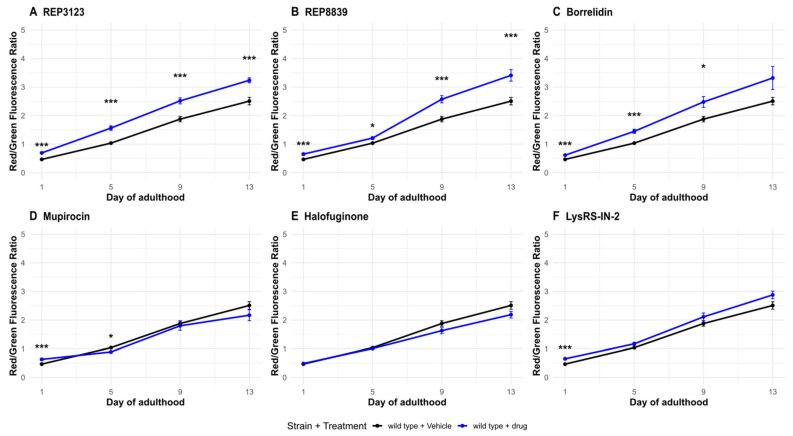
Autophagic flux increases following some tRNA synthetase inhibitor treatments. (**A**) A total of 40 μM REP3123 significantly increases the ratio of Red/Green fluorescence in wild-type drug-treated *C. elegans* (blue) compared to the vehicle-treated group (black) on all days tested (1, 5, 9, and 13). (**B**) A total of 60 μM REP8839 significantly increases the ratio of Red/Green fluorescence in wild-type drug-treated *C. elegans* (blue) compared to the vehicle-treated group (black) on all days tested (1, 5, 9, and 13). (**C**) A total of 75 μM borrelidin significantly increases the ratio of Red/Green fluorescence in wild-type drug-treated *C. elegans* (blue) compared to the vehicle-treated group (black) on days 1, 5, and 9. (**D**) A total of 550 μM mupirocin significantly increases the ratio of Red/Green fluorescence in wild-type drug-treated *C. elegans* (blue) compared to the vehicle-treated group (black) on day 1. (**E**) A total of 1 μM halofuginone does not significantly increase autophagic flux on any day tested. (**F**) A total of 10 μM Lys-RS-In-2 significantly increases the ratio of Red/Green fluorescence in wild-type drug-treated *C. elegans* (blue) compared to the vehicle-treated group (black) on day 1. Vehicle = DMSO. * *p* < 0.05, *** *p* < 0.001. *p* values calculated with Student’s *t*-test with Bonferroni correction (*n* = 4 comparisons per timepoint).

**Figure 6:**
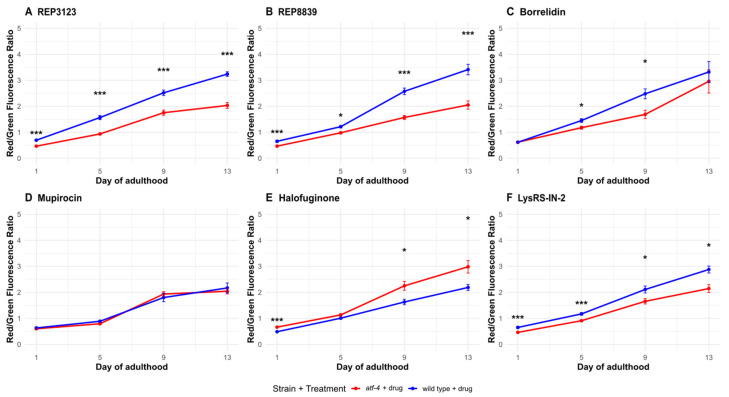
Autophagic flux increases following some tRNA synthetase inhibitor treatments partially depend on *atf-4(ok576)*. (**A**) A total of 40 μM REP3123 significantly increases the ratio of Red/Green fluorescence in wild-type drug-treated *C. elegans* (blue) compared to the *atf-4(ok576)* drug-treated group (red) on all days tested (1, 5, 9, and 13). (**B**) A total of 60 μM REP8839 significantly increases the ratio of Red/Green fluorescence in wild-type drug-treated *C. elegans* (blue) compared to the *atf-4(ok576)* drug-treated group (red) on all days tested (1, 5, 9, and 13). (**C**) A total of 75 μM borrelidin significantly increases the ratio of Red/Green fluorescence in wild-type drug-treated *C. elegans* (blue) compared to the *atf-4(ok576)* drug-treated group (red) on days 5 and 9. (**D**) A total of 550 μM mupirocin did not significantly increase the ratio of Red/Green fluorescence in wild-type drug-treated *C. elegans* (blue) compared to the *atf-4(ok576)* drug-treated group (red) on any day tested. (**E**) A total of 1 μM halofuginone significantly increases the ratio of Red/Green fluorescence in *atf-4(ok576)* drug-treated *C. elegans* (red) compared to the wild-type drug-treated group (blue) on days 1, 9, and 13. (**F**) A total of 10 μM Lys-RS-In-2 significantly increases the ratio of Red/Green fluorescence in wild-type drug-treated *C. elegans* (blue) compared to the *atf-4(ok576)* drug-treated group (red) on all days tested (1, 5, 9, and 13). Vehicle = DMSO. (mean thrashing, *n*) * *p* < 0.05, *** *p* < 0.001. *p* values calculated with Student’s *t*-test with Bonferroni correction (*n* = 4 comparisons per timepoint). *p* values calculated with Student’s *t*-test with Bonferroni correction (*n* = 4 comparisons per timepoint). The *atf-4(ok576)* + vehicle control was omitted here for clarity, but is shown in Supplementary Figure S1, where all conditions are displayed side by side.

To further investigate this, we examined autophagy in *C. elegans* using a dual-color fluorescent reporter strain, MAH215, which expresses tandem-tagged GFP and mCherry fused to LGG-1, the *C. elegans* homolog of human LC3 [24]. We found that treatment with tRNA synthetase inhibitors leads to a substantial increase in autophagic activity in *C. elegans* at the same concentrations that extend lifespan (Figure 5). This increase in autophagy was dependent in part on the presence of *atf-4* (Figure 6), suggesting a potential link between ATF-4 upregulation, autophagy, and lifespan extension.

Consistent with these findings, quantitative analysis of tagged LGG-1 puncta revealed a marked elevation in autophagolysosome formation in tRNA synthetase inhibitor-treated animals, particularly during mid-to-late adulthood when autophagic flux typically declines. The use of the dual GFP::mCherry tag allowed us to distinguish between autophagosomes and autolysosomes, and our data indicate enhanced autophagic flux rather than simple accumulation of autophagic intermediates. Importantly, for some of these compounds, no increase in autophagic activity was observed in *atf-4(ok576)* mutants treated with the same doses, supporting a model in which ATF-4 acts upstream of autophagy induction under these conditions. Notably, not all tRNA synthetase inhibitors produced a significant increase in autophagic flux. This variability likely reflects compound-specific mechanisms, as each inhibitor targets a distinct aminoacyl-tRNA synthetase with unique cellular roles and stress response profiles. Such differences in target enzyme abundance, localization, or affinity could lead to varying levels of uncharged tRNA accumulation and downstream activation of the ATF-4 pathway. These findings suggest that the induction of autophagy by tRNA synthetase inhibitors is not universal but instead depends on the degree and nature of translational stress imposed by each compound.

Taken together, these results suggest that autophagy acts as one of several effector pathways downstream of ATF-4 in response to tRNA synthetase inhibition. However, not all compounds required ATF-4 for their beneficial effects, indicating that additional or parallel signaling pathways likely contribute to the observed improvements in lifespan and motility. The induction of autophagy likely contributes to improved proteostasis and cellular maintenance during aging, thereby promoting both the increased lifespan and healthspan seen following treatments with these same concentrations of tRS inhibitors. Thus, tRNA synthetase inhibitors appear to elicit a conserved, adaptive response involving ATF-4 and autophagy that supports healthy aging.

## 4. Discussion

Our findings identify four novel tRNA synthetase inhibitors that can robustly extend lifespan in *C. elegans.* These inhibitors target different tRNA synthetases, consistent with our model in which the accumulation of many distinct uncharged tRNAs should lead to translational upregulation of ATF-4 and increased transcription of its targets. Combined with our work showing similar effects of these compounds on ATF4 in mammalian cells [16], and the work of others showing upregulation of ATF4 in long-lived mice [25,26], these results support the idea that tRNA synthetase inhibition may engage conserved stress response pathways relevant to aging. Notably, several of these compounds also improved healthspan in *C. elegans*, as assessed by age-dependent declines in thrashing behavior at the same doses that extended lifespan, raising the possibility that tRNA synthetase inhibition could delay functional decline during aging.

Two of the compounds identified in this study—REP3123 and REP8839—target methionyl tRNA synthetase, while LysRS-In-2 and halofuginone inhibit lysyl and prolyl-tRNA synthetases, respectively. All four significantly increase lifespan in wild-type *C. elegans* (Figure 1). This effect was completely abolished in *atf-4(ok576)* mutants (Figure 2), confirming that ATF-4 activation is necessary for the observed longevity benefits for the tested compounds. These data support a possible model in which tRNA synthetase inhibition engages an adaptive transcription factor response, ultimately leading to increased organism survival [8,21].

Halofuginone increased lifespan in the wild type, yet showed no wild-type autophagic flux increase and, in fact, showed a relative flux elevation in *atf-4(ok576)* animals at several timepoints. Given halofuginone’s known tolerability limits and reported off-target effects [27,28], it is possible that this compound may trigger compensatory, ATF-4-independent stress responses at the tested maximally lifespan-extending dose.

Interestingly, some tRNA synthetase inhibitors appeared to reduce lifespan in *atf-4(ok576)* mutants, possibly suggesting increased sensitivity to translational stress in *atf-4(ok576)*. Loss of *atf-4* compromises the ability to upregulate stress-responsive and proteostasis-related genes, which may render animals less tolerant to inhibitors that partially suppress protein synthesis. Because the concentrations used were optimized for maximal lifespan extension in the wild type, these same doses may approach the toxicity threshold in the potentially more sensitive *atf-4* background. Thus, the apparent toxicity in *atf-4* mutants could reflect a combination of dose-dependent effects and reduced stress resilience rather than a fundamentally distinct mechanism of action.

The degree of *atf-4* dependence varied across the inhibitors tested in our autophagy and thrashing data. This variability could arise from several factors. Although all compounds ultimately limit aminoacyl-tRNA synthetase activity, they act on distinct enzymes that may differ in expression level, substrate specificity, or cellular localization. As a result, each inhibitor could produce a unique pattern of uncharged tRNA accumulation and thus distinct activation kinetics of the AAR pathway. Additionally, some inhibitors—such as halofuginone, which is near its tolerability limit—may engage other stress response pathways, including the unfolded protein response or oxidative stress signaling, and do so in an *atf-4*-independent manner. Differences in compound stability, uptake, or metabolism may also contribute to apparent differences in *atf-4* dependence. Together, these factors could explain the varying *atf-4* interactions with healthspan and autophagy phenotypes observed across this drug panel.

Although our results demonstrate that loss of *atf-4* suppresses lifespan and partially suppresses autophagy responses to tRNA synthetase inhibition, we did not directly measure ATF-4 activation in this study. Future work employing ATF-4 reporter strains or transcriptional analysis of known ATF-4 target genes will be essential to confirm pathway activation and to define the broader transcriptional program engaged by these compounds.

Previous work in MEFs showed that tRNA synthetase inhibition leads to increased ATF4 and also to ATF4-dependent increased autophagy [16]. Here, using the autophagy reporter strain MAH215, we demonstrate that autophagy is markedly increased in *C. elegans* following treatment with lifespan-extending doses of some of the same tRNA synthetase inhibitors (Figure 5). Importantly, this autophagic response is in some cases partially ATF-4-dependent (Figure 6), suggesting a possible mechanistic link between ATF-4 activation, autophagy, and longevity. These findings provide in vivo support for a model in which ATF-4-driven enhancement of autophagy could be an effector of the longevity response to translational stress.

That said, the observation that only a subset of compounds robustly increased autophagic flux indicates that this pathway is not uniformly activated by all tRNA synthetase inhibitors at the lifespan-extending doses tested here. The extent of autophagy induction may depend on differences in enzyme target class, compound potency, or the specific stress response pathways engaged. Some inhibitors may primarily activate alternative adaptive mechanisms, such as unfolded protein or oxidative stress responses, rather than canonical ATF-4-driven autophagy.

While our data indicate that tRNA synthetase inhibitors enhance autophagic flux and that this response is partially *atf-4*-dependent, we cannot conclude any cause–effect relationship between increased autophagic flux and increased lifespan in these contexts based on these data alone. Directly testing this hypothesis by blocking autophagy is challenging in part because autophagy-deficient mutants, such as *bec-1*, *atg-7*, or *lgg-1,* exhibit shortened lifespans, developmental arrest, and compromised health even under control conditions [22,29], although chemical inhibitors such as bafilomycin could be utilized in future studies. Nevertheless, the parallel increases in autophagic flux and lifespan, and the loss of flux induction in the *atf-4(ok576)* background, are consistent with the possibility that autophagy might contribute functionally to the adaptive response elicited by tRNA synthetase inhibition.

Beyond lifespan, we also assessed healthspan through age-related declines in motility using the thrashing assay. Treatment with some tRNA synthetase inhibitors resulted in a sustained increase in thrashing behavior throughout aging, suggesting improved overall vitality in later life (Figure 3). Strikingly, as with lifespan and autophagy, these improvements seemed to be partially dependent on the presence of *atf-4* (Figure 4) in some of the drug treatments, further reinforcing its role as a central mediator of both longevity and functional health [30]. It should be noted that our healthspan analysis was based solely on thrashing behavior, which primarily reflects neuromuscular function. While this is a commonly used metric in *C. elegans* aging studies, other measures of healthspan, such as reproductive capacity, pharyngeal pumping, or stress resistance, may not always correlate directly with motility. Future work employing multiple independent healthspan metrics will be important to determine the extent to which the benefits of tRNA synthetase inhibition extend across physiological systems.

One limitation of this study is that thrashing and autophagic flux measurements were obtained from single synchronized cohorts per experiment, with multiple individual animals assessed within each strain × condition × day, rather than from independent biological replicates performed across separate experimental days. As a result, these datasets capture within-cohort variability but do not fully account for between-cohort variation. Replication across independent synchronized populations will be important in future work to confirm the robustness and generalizability of these healthspan and autophagy phenotypes.

Together, these results establish a clear and compelling link between tRNA synthetase inhibition, ATF-4 activation, and healthy aging. By extending these findings across multiple inhibitors and phenotypic readouts, we propose that tRNA synthetase inhibition acts as a broadly effective strategy to trigger conserved stress response pathways that enhance longevity and quality of life. Future work will be needed to explore the translational relevance of these findings in mammalian systems.

In particular, identifying specific ATF-4 target genes required for the observed longevity benefits may provide insights into the mechanisms by which this transcription factor influences aging. Additionally, the conservation of these effects across species raises the possibility of developing targeted therapeutics that mimic the effects of tRNA synthetase inhibition. Small molecules that selectively modulate the ATF-4 pathway or its downstream effectors could therefore represent a novel class of interventions aimed at promoting healthy aging. Finally, longitudinal studies in mammalian models will be crucial to determine whether similar improvements in healthspan and lifespan can be achieved in complex organisms and to evaluate the safety and efficacy of long-term modulation of this pathway.

## 5. Conclusions

In summary, our study identifies multiple tRNA synthetase inhibitors that extend both lifespan and healthspan in *C. elegans* through a conserved and partially ATF-4–dependent mechanism. These findings not only reinforce the role of ATF-4 as a critical mediator of the cellular response to translational stress but also highlight autophagy as a likely effector of this longevity pathway. By linking tRNA synthetase inhibition to increased vitality and extended lifespan, this work lays the foundation for exploring ATF-4 activation as a therapeutic strategy for promoting healthy aging in higher organisms.

## Data Availability

The original contributions presented in this study are included in the article/Supplementary Material. Further inquiries can be directed to the corresponding author.

## Supplementary Materials

The following supporting information can be downloaded at: https://www.mdpi.com/article/10.3390/biom16010073/s1, Figure S1: Autophagic flux increases following some tRNA synthetase inhibitor treatments in a manner that is partially dependent on atf-4(ok576).; Table S1: Autophagic flux *p*-values. Vehicle = DMSO. *p* values calculated with Student’s *t*-test with Bonferroni correction (*n* = 4 comparisons per timepoint).; Table S2: Thrashing *p*-values. Vehicle = DMSO. *p* values calculated with Student’s *t*-test with Bonferroni correction (*n* = 7 comparisons per drug panel).; Table S3: Summary of mean lifespan values for *C. elegans* wild-type (N2) and *atf-4(ok576)* animals treated with tRNA synthetase inhibitors. Each dataset represents one independent experiment with approximately 100-250 worms per condition. Values indicate mean lifespan (days) and the number of animals analyzed (*n*). Statistical significance was determined using the log-rank (Mantel-Cox) test implemented in R. These data correspond to Figure 1 and Figure 2 in the main text. The *atf-4* condition’s significance indicates groups that were significantly shorter lived than the vehicle condition.

## Abbreviations

The following abbreviations are used in this manuscript:

ATF-4	Activating transcription factor 4
tRNA	Transfer RNA
TRS	tRNA synthetase
AAR	Amino acid stress response
eIF-2	Eukaryotic initiation factor 2
NGM	Nematode growth media
GCN2	General control nonderepressible 2
MEFs	Mouse embryonic fibroblasts
GCN4	General control nonderepressible 4
LC3	Microtubule-associated protein 1 light chain 3

## References

[1] Klass M.R. (1983). A Method for the Isolation of Longevity Mutants in the Nematode *Caenorhabditis elegans* and Initial Results. Mech. Ageing Dev..

[2] Friedman D.B., Johnson T.E. (1988). A Mutation in the Age-1 Gene in *Caenorhabditis elegans* Lengthens Life and Reduces Hermaphrodite Fertility. Genetics.

[3] Kenyon C., Chang J., Gensch E., Rudner A., Tabtiang R. (1993). A *C. elegans* Mutant That Lives Twice as Long as Wild Type. Nature.

[4] Kimura K.D., Tissenbaum H.A., Liu Y., Ruvkun G. (1997). Daf-2, an Insulin Receptor-like Gene That Regulates Longevity and Diapause in *Caenorhabditis elegans*. Science.

[5] Lai C.-H., Chou C.-Y., Ch’ang L.-Y., Liu C.-S., Lin W. (2000). Identification of Novel Human Genes Evolutionarily Conserved in *Caenorhabditis elegans* by Comparative Proteomics. Genome Res..

[6] Robbins C.E., Patel B., Sawyer D.L., Wilkinson B., Kennedy B.K., McCormick M.A. (2022). Cytosolic and Mitochondrial tRNA Synthetase Inhibitors Increase Lifespan in a *GCN4/Atf-4*-Dependent Manner. iScience.

[7] Ramirez M., Wek R.C., De Aldana C.R., Jackson B.M., Freeman B., Hinnebusch A.G. (1992). Mutations Activating the Yeast eIF-2a Kinase GCN2: Isolation of Alleles Altering the Domain Related to Histidyl-tRNA Synthetases. Mol. Cell. Biol..

[8] Hinnebusch A.G., Natarajan K. (2002). Gcn4p, a Master Regulator of Gene Expression, Is Controlled at Multiple Levels by Diverse Signals of Starvation and Stress. Eukaryot. Cell.

[9] Ameri K., Harris A.L. (2008). Activating Transcription Factor 4. Int. J. Biochem. Cell Biol..

[10] Lu P.D., Harding H.P., Ron D. (2004). Translation Reinitiation at Alternative Open Reading Frames Regulates Gene Expression in an Integrated Stress Response. J. Cell Biol..

[11] Brenner S. (1974). The Genetics of *Caenorhabditis elegans*. Genetics.

[12] Hsin H., Kenyon C. (1999). Signals from the Reproductive System Regulate the Lifespan of *C. elegans*. Nature.

[13] Meneely P.M., Dahlberg C.L., Rose J.K. (2019). Working with Worms: *Caenorhabditis elegans* as a Model Organism. Curr. Protoc. Essent. Lab. Tech..

[14] Kaplan E.L., Meier P. (1958). Nonparametric Estimation from Incomplete Observations. J Amer Stat. Assn.

[15] Student A. (1908). The Probably Error of a Mean. Biometrika.

[16] Mariner B.L., Rodriguez A.S., Heath O.C., McCormick M.A. (2024). Induction of Proteasomal Activity in Mammalian Cells by Lifespan-Extending tRNA Synthetase Inhibitors. GeroScience.

[17] Cinader B. (1989). Aging, Evolution and Individual Health Span: Introduction. Genome.

[18] Globerson A., Barzilai N. (2005). The Voyage to Healthy Longevity: From Experimental Models to the Ultimate Goal. Mech. Ageing Dev..

[19] Carter C.S., Hofer T., Seo A.Y., Leeuwenburgh C. (2007). Molecular Mechanisms of Life- and Health-Span Extension: Role of Calorie Restriction and Exercise Intervention. Appl. Physiol. Nutr. Metab. Physiol. Appl. Nutr. Metab..

[20] Koopman M., Seinstra R.I., Nollen E.A.A. (2019). *C. elegans* as a Model for Synucleinopathies and Other Neurodegenerative Diseases: Tools and Techniques. Methods Mol. Biol. Clifton NJ.

[21] Hinnebusch A.G. (2005). Translational Regulation of GCN4 and the General Amino Acid Control of Yeast*. Annu. Rev. Microbiol..

[22] Hansen M., Chandra A., Mitic L.L., Onken B., Driscoll M., Kenyon C. (2008). A Role for Autophagy in the Extension of Lifespan by Dietary Restriction in *C. elegans*. PLoS Genet..

[23] Mariner B.L., Felker D.P., Cantergiani R.J., Peterson J., McCormick M.A. (2023). Multiomics of GCN4-Dependent Replicative Lifespan Extension Models Reveals Gcn4 as a Regulator of Protein Turnover in Yeast. Int. J. Mol. Sci..

[24] Chang J.T., Kumsta C., Hellman A.B., Adams L.M., Hansen M. (2017). Spatiotemporal Regulation of Autophagy during *Caenorhabditis elegans* Aging. ELife.

[25] Li W., Li X., Miller R.A. (2014). ATF4 Activity: A Common Feature Shared by Many Kinds of Slow-Aging Mice. Aging Cell.

[26] Li W., Miller R.A. (2015). Elevated ATF4 Function in Fibroblasts and Liver of Slow-Aging Mutant Mice. J. Gerontol. A. Biol. Sci. Med. Sci..

[27] Bampidis V., Azimonti G., Bastos M.d.L., Christensen H., Dusemund B., Fašmon Durjava M., Kouba M., López-Alonso M., López Puente S., EFSA Panel on Additives and Products or Substances used in Animal Feed (FEEDAP) (2020). Safety and Efficacy of STENOROL® (Halofuginone Hydrobromide) as a Feed Additive for Chickens for Fattening and Turkeys. EFSA J..

[28] Pines M., Spector I. (2015). Halofuginone—The Multifaceted Molecule. Molecules.

[29] Meléndez A., Tallóczy Z., Seaman M., Eskelinen E.-L., Hall D.H., Levine B. (2003). Autophagy Genes Are Essential for Dauer Development and Life-Span Extension in *C. elegans*. Science.

[30] Rousakis A., Vlassis A., Vlanti A., Patera S., Thireos G., Syntichaki P. (2013). The General Control Nonderepressible-2 Kinase Mediates Stress Response and Longevity Induced by Target of Rapamycin Inactivation in *Caenorhabditis elegans*. Aging Cell.

