# Supplementary material for: Novel tRNA Synthetase Inhibitors Increase Healthspan, Lifespan, and Autophagic Flux in C. elegans
Source: Biomolecules. 2026 Jan 1;16(1):73. doi: 10.3390/biom16010073 (PMC12839378; doi:10.3390/biom16010073)
Supplement: Supplementary file 1 [file biomolecules-16-00073-s001.zip › biomolecules-3934903-supplementary.pdf]

biomolecules

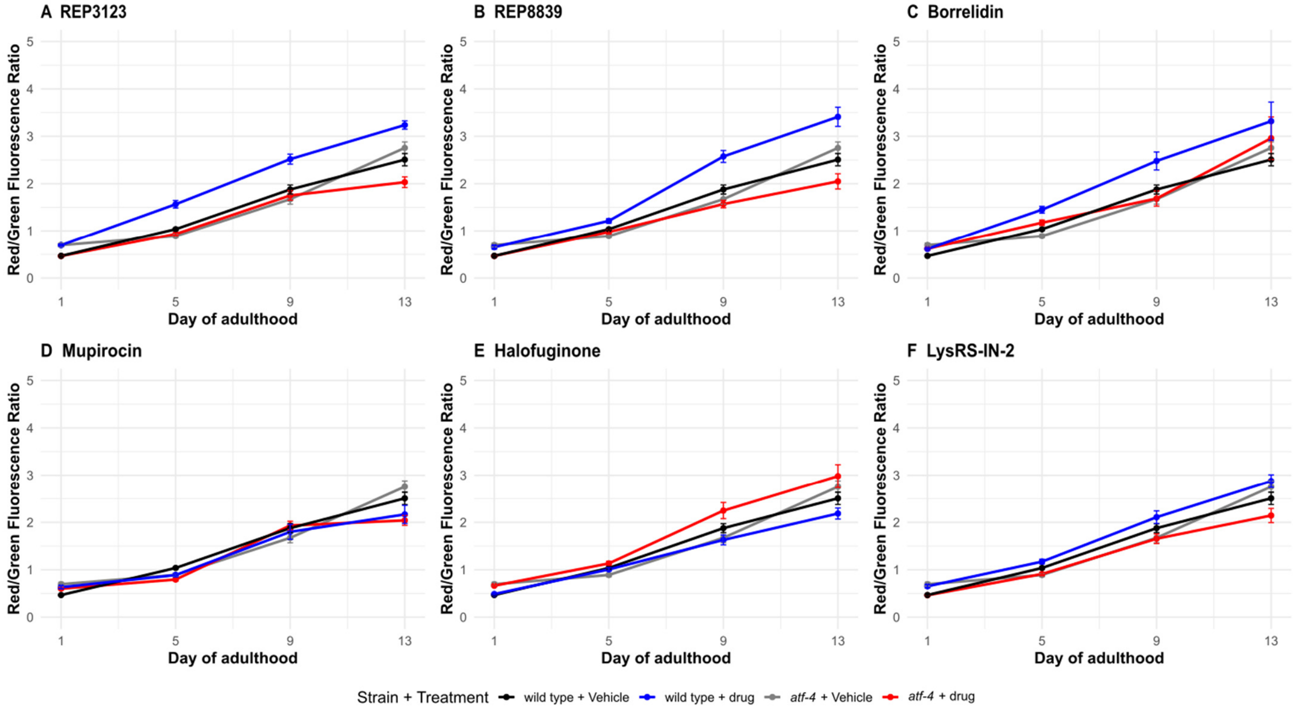

Supplemental Figure S1. Autophagic flux increases following some tRNA synthetase inhibitor treatments in a manner that is partially dependent on *atf-4(ok576)*. (**A**) 40 µM REP3123 significantly increases the ratio of Red/Green fluorescence in wild type drug treated *C. elegans* (blue) compared to the *atf-4(ok576)* drug treated group (red) on all days tested (1, 5, 9, and 13). (**B**) 60 µM REP8839 significantly increases the ratio of Red/Green fluorescence in wild type drug treated *C. elegans* (blue) compared to the *atf-4(ok576)* drug treated group (red) on all days tested (1, 5, 9, and 13). (**C**) 75 µM Borrelidin significantly increases the ratio of Red/Green fluorescence in wild type drug treated *C. elegans* (blue) compared to the *atf-4(ok576)* drug treated group (red) on days 5 and 9. (**D**) 550 µM Mupirocin did not significantly increases the ratio of Red/Green fluorescence in wild type drug treated *C. elegans* (blue) compared to the *atf-4(ok576)* drug treated group (red) on any day tested. (**E**) 1 µM Halofuginone significantly increases the ratio of Red/Green fluorescence in *atf-4(ok576)* drug treated *C. elegans* (red) compared to the wild type drug treated group (blue) on days 1, 9, and 13. (**F**) 10 µM Lys-RS-In-2 significantly increases the ratio of Red/Green fluorescence in wild type drug treated *C. elegans* (blue) compared to the *atf-4(ok576)* drug treated group (red) on all days tested (1, 5, 9 and 13). Vehicle = DMSO. P values calculated with **Student's t-test** with **Bonferroni correction** (n = 4 comparisons per timepoint).

 https://doi.org/10.3390/biom16010073

Table S1. Autophagic flux p-values. Vehicle = DMSO. P values calculated with **Student's t-test** with **Bonferroni correction** (n = 4 comparisons per timepoint).

REP3123

**Day**	wild type drug vs wild type vehicle	wild type drug vs *atf-4(ok576)* drug	wild type vehicle vs *atf-4(ok576)* vehicle	*atf-4(ok576)* drug vs *atf-4(ok576)* vehicle
**1**	0.0000	0.0000	0.0003	0.0003
**5**	0.0000	0.0000	0.0101	1.0000
**9**	0.0005	0.0000	0.6374	1.0000
**13**	0.0004	0.0000	0.7352	0.0008

REP8839

**Day**	wild type drug vs wild type vehicle	wild type drug vs *atf-4(ok576)* drug	wild type vehicle vs *atf-4(ok576)* vehicle	*atf-4(ok576)* drug vs *atf-4(ok576)* vehicle
**1**	0.0028	0.0026	0.0003	0.0003
**5**	0.0154	0.0046	0.0101	0.4801
**9**	0.0006	0.0000	0.6374	1.0000
**13**	0.0039	0.0001	0.7352	0.0077

Borrelidin

**Day**	wild type drug vs wild type vehicle	wild type drug vs *atf-4(ok576)* drug	wild type vehicle vs *atf-4(ok576)* vehicle	*atf-4(ok576)* drug vs *atf-4(ok576)* vehicle
**1**	0.0004	1.0000	0.0003	0.5251
**5**	0.0004	0.0295	0.0101	0.0022
**9**	0.0467	0.0172	0.6374	1.0000
**13**	0.3173	1.0000	0.7352	1.0000

Mupirocin

**Day**	wild type drug vs wild type vehicle	wild type drug vs *atf-4(ok576)* drug	wild type vehicle vs *atf-4(ok576)* vehicle	*atf-4(ok576)* drug vs *atf-4(ok576)* vehicle
**1**	0.0018	1.0000	0.0003	0.2465
**5**	0.0187	0.3597	0.0101	0.2671
**9**	1.0000	1.0000	0.6374	0.2808
**13**	0.6157	1.0000	0.7352	0.0006

Halofuginone

**Day**	wild type drug vs wild type vehicle	wild type drug vs *atf-4(ok576)* drug	wild type vehicle vs *atf-4(ok576)* vehicle	*atf-4(ok576)* drug vs *atf-4(ok576)* vehicle
**1**	1.0000	0.0000	0.0003	1.0000
**5**	1.0000	0.1404	0.0101	0.0010
**9**	0.3545	0.0200	0.6374	0.0341
**13**	0.3096	0.0311	0.7352	1.0000

LysRS-IN-2

**Day**	wild type drug vs wild type vehicle	wild type drug vs *atf-4(ok576)* drug	wild type vehicle vs *atf-4(ok576)* vehicle	*atf-4(ok576)* drug vs *atf-4(ok576)* vehicle
**1**	0.0006	0.0005	0.0003	0.0003
**5**	0.1032	0.0010	0.0101	1.0000
**9**	0.6747	0.0470	0.6374	1.0000
**13**	0.2374	0.0044	0.7352	0.0167

https://doi.org/10.3390/biom16010073

Table S2. Thrashing p-values. Vehicle = DMSO. P values calculated with Student's t-test with Bonferroni correction (n = 7 comparisons per drug panel).

REP3123

Day	wild type drug vs wild type vehicle	wild type drug vs *atf-4(ok576)* drug	wild type vehicle vs *atf-4(ok576)* vehicle	*atf-4(ok576)* drug vs *atf-4(ok576)* vehicle
**1**	0.6678	0.4270	0.6066	0.0206
**4**	0.0093	0.8090	0.0031	0.8190
**7**	0.0000	0.0002	0.0864	0.0000
**10**	0.0058	0.2582	0.0111	0.0000
**11**	0.0008	0.0122	0.4176	0.0698
**13**	0.0144	0.2829	0.3934	0.0025
**17**	0.0412	0.0016	0.1643	0.0036

REP8839

Day	wild type drug vs wild type vehicle	wild type drug vs *atf-4(ok576)* drug	wild type vehicle vs *atf-4(ok576)* vehicle	*atf-4(ok576)* drug vs *atf-4(ok576)* vehicle
**1**	0.4777	0.8812	0.6066	0.1129
**4**	0.3000	0.9803	0.0031	0.1134
**7**	0.0046	0.0124	0.0864	0.6944
**10**	0.0223	0.0014	0.0111	0.7993
**11**	0.0109	1.0000	0.4176	0.0050
**13**	0.0002	0.6051	0.3934	0.0000
**17**	0.2382	0.7513	0.1643	0.6072

Borrelidin

Day	wild type drug vs wild type vehicle	wild type drug vs *atf-4(ok576)* drug	wild type vehicle vs *atf-4(ok576)* vehicle	*atf-4(ok576)* drug vs *atf-4(ok576)* vehicle
**1**	0.5924	0.4969	0.6066	0.7941
**4**	0.0272	0.9544	0.0031	0.4928
**7**	0.0008	0.0033	0.0864	0.0067
**10**	0.1257	0.1895	0.0111	0.0000
**11**	0.0236	0.0123	0.4176	0.1219
**13**	0.1141	0.1277	0.3934	0.2709
**17**	1.0000	0.4217	0.1643	0.6851

Halofuginone

Day	wild type drug vs wild type vehicle	wild type drug vs *atf-4(ok576)* drug	wild type vehicle vs *atf-4(ok576)* vehicle	*atf-4(ok576)* drug vs *atf-4(ok576)* vehicle
**1**	0.5427	0.4188	0.6066	0.3814
**4**	0.7231	0.1599	0.0031	0.0699
**7**	0.7440	0.7629	0.0864	0.1240
**10**	0.4096	0.4026	0.0111	0.1589
**11**	0.3254	0.7918	0.4176	0.9790
**13**	0.3603	0.1671	0.3934	0.3482
**17**	0.6371	0.4390	0.1643	0.0927

LysRS-IN-2

https://doi.org/10.3390/biom16010073

**Day**	wild type drug vs wild type vehicle	wild type drug vs *atf-4(ok576)* drug	wild type vehicle vs *atf-4(ok576)* vehicle	*atf-4(ok576)* drug vs *atf-4(ok576)* vehicle
**1**	0.2807	0.4837	0.6066	0.9859
**4**	0.3813	0.0622	0.0031	0.0995
**7**	0.3924	0.0994	0.0864	0.1455
**10**	0.2918	0.0692	0.0111	0.9725
**11**	0.0128	0.0191	0.4176	0.5787
**13**	0.5429	0.4938	0.3934	0.3450
**17**	0.1847	0.7808	0.1643	0.4938

Mupirocin

**Day**	wild type drug vs wild type vehicle	wild type drug vs *atf-4(ok576)* drug	wild type vehicle vs *atf-4(ok576)* vehicle	*atf-4(ok576)* drug vs *atf-4(ok576)* vehicle
**1**	0.9064	0.0312	0.6066	0.0056
**4**	0.0466	0.0000	0.0031	0.6869
**7**	0.0170	0.2653	0.0864	0.0000
**10**	0.0000	0.2696	0.0111	0.0000
**11**	0.0000	0.8066	0.4176	0.0001
**13**	0.0251	0.3683	0.3934	0.0010
**17**	0.0056	0.0776	0.1643	0.3423

Table S3. Summary of mean lifespan values for C. elegans wild-type (N2) and atf-4(ok576) animals treated with tRNA synthetase inhibitors. Each dataset represents one independent experiment with approximately 100-250 worms per condition. Values indicate mean lifespan (days) and the number of animals analyzed (n). Statistical significance was determined using the log-rank (Mantel-Cox) test implemented in R. These data correspond to Figures 1 and 2 in the main text. The atf-4 condition's significance indicates groups that were significantly shorter lived than the vehicle condition

Strain	Compound	Concentration (µM)	Mean Lifespan (days)	n (worms)	p vs Vehicle	Statistical Test
atf-4(ok576)	Vehicle (DMSO)	–	23.2	226	–	Log-rank (Mantel–Cox)
	LysRS-IN-2	10	18.4	156	p=1.85E-13	Log-rank
	REP8839	60	20.8	159	p=0.063	Log-rank
	Halofuginone	1	24.6	175	p=0.0026	Log-rank
	REP3123	40	20.5	129	p=0.0089	Log-rank
Wild type (N2)	Vehicle (DMSO)	–	22.8	123	–	Log-rank
	LysRS-IN-2	10	25.5	144	p= 0.001	Log-rank
	REP8839	60	29.3	146	p=4.31E-13	Log-rank

https://doi.org/10.3390/biom16010073

Halofugi-none	1	28.9	172	p=2.81E-14	Log-rank
REP3123	40	21.7	111	p=9.69E-07	Log-rank

https://doi.org/10.3390/biom16010073
